# Psychometric evaluation of the Chinese version of Mindful Eating Scale in Chinese adults: a translation and validation study

**DOI:** 10.3389/fpubh.2026.1895623

**Published:** 2026-09-09

**Authors:** Wenguang Xie, Qian Huang, Jian Kang, Wenhao Zhang, Yidan Liu, Ping Zhou, Chao Zhang

**Affiliations:** 1 The 2nd Affiliated Hospital, Jiangxi Medical College, Nanchang University, Nanchang, China; 2 School of Nursing, Jiangxi Medical College, Nanchang University, Nanchang, China; 3 Xinjiang Hetian College, Hetian, China; 4 Department of Cardiology One, Hongqi Hospital Affiliated to Mudanjiang Medical University, Mudanjaing, China

**Keywords:** mindful eating, nutrition, reliability, scale, validity

## Abstract

**Background:**

Mindful eating serves as a critical behavioral strategy for promoting healthy dietary practices and enhancing mental wellbeing. However, there is currently a notable absence of a rigorously validated mindful eating assessment tool that is applicable to the general population in China. This study aims to translate the Mindful Eating Scale (Mind-Eat Scale) into Chinese and to validate the psychometric properties of the Chinese version within the general population of China.

**Methods:**

A convenience sampling method was employed to recruit a total of 346 adults from three communities across three provinces in China: Jiangxi Province, Xinjiang Uygur Autonomous Region, and Heilongjiang Province. The Brislin translation model was employed for forward-backward translation. A multidisciplinary expert panel (*n* = 7) conducted cross-cultural adaptation using the Delphi method, followed by a pilot test among 20 adults to confirm item clarity and comprehensibility. The reliability of the scale was assessed through internal consistency reliability, split-half reliability, and test–retest reliability. The validity of the scale was evaluated through both content validity and structural validity.

**Results:**

The Chinese version of the mind-eat scale comprises six dimensions: awareness, non-reactivity, openness, gratitude, non-judgment, and hunger/satiety, totaling 24 items. The overall Cronbach’s *α* coefficient for the scale was 0.927, with coefficients for the six dimensions ranging from 0.757 to 0.864. The split-half reliability was found to be 0.895, while the test–retest reliability was 0.862. Regarding content validity, the I-CVI values ranged from 0.857 to 1.000, and the S-CVI was 0.964. Exploratory factor analysis revealed six factors with eigenvalues greater than 1, accounting for a cumulative variance explained rate of 69.135%. Confirmatory factor analysis demonstrated a good model fit with χ^2^/df = 2.273, RMSEA = 0.078, CFI = 0.904, TLI = 0.908, IFI = 0.906, and PNFI = 0.706.

**Conclusion:**

The Mind-Eat Scale exhibits satisfactory psychometric properties within a community-based convenience sample of Chinese adults, establishing it as a preliminary instrument for assessing mindful eating levels in this specific population. However, further validation in more diverse and clinical populations is warranted before broader application.

## Introduction

Mindfulness is defined as “the awareness that arises when one intentionally focuses attention on the present moment and observes the unfolding of the present experience in an unjudgmental way” (1). This concept originated from the Buddhist meditation tradition and was later integrated into modern medicine and mental health fields as a supplementary approach to assist patients in coping with stress, pain, and functional impairments (2). In recent years, the application of mindfulness in dietary behavior—specifically termed “mindful eating”—has garnered increasing attention and has emerged as a significant behavioral strategy for promoting healthy eating and enhancing mental health (3). Although the concept of mindful eating lacks a unified definition, there is a consensus in the literature regarding its core principles: it entails engaging all senses to focus on the eating experience, observing emotions and physical reactions without judgment before, during, and after eating, cultivating awareness of hunger and fullness, and minimizing automatic responses to external eating cues (3). Research indicates that this behavioral strategy positively influences food intake by enhancing the perception of internal eating signals and diminishing emotional and external eating triggers (4).

Mindful eating is intricately linked to various dimensions of health outcomes. Research indicates that individuals who practice mindful eating exhibit healthier dietary behaviors, maintain lower body weight, and possess more favorable cardiovascular biomarkers (5, 6). Specifically, mindful eating is positively correlated with enhanced dietary quality, characterized by increased consumption of fruits, vegetables, and whole grains, alongside a reduced intake of ultra-processed foods and sugary beverages (7). Psychologically, mindful eating is significantly associated with lower levels of emotional eating, uncontrolled eating, depression, anxiety, and stress (8, 9). Additionally, it has been shown to effectively mitigate binge eating behaviors and tendencies toward food addiction (10, 11). These findings suggest that mindful eating not only influences an individual’s nutritional status but also profoundly impacts brain health and mental wellbeing by regulating emotional and cognitive processes. From a neuroscientific viewpoint, mindful eating can recalibrate the reward processing mechanisms, enhance neural cognitive flexibility, and promote stress regulation (12). Considering the critical role of nutrition in preventing non-communicable diseases—where one in five deaths globally could be averted through improved dietary quality (13)—and the low adherence to traditional dietary advice, which often overlooks psychological factors (9), mindful eating emerges as an effective strategy for fostering healthy eating habits (7). Notably, this concept has garnered recognition from authoritative public health institutions, as demonstrated by the updated Canadian Food Guide, which explicitly advocates for mindful eating and encourages individuals to focus on their eating experiences while attuning to hunger and satiety signals (14).

With the rapid advancement of research in the field of mindful eating, reliable measurement tools have become essential for conducting quantitative studies. Over the past decade, researchers have developed various multidimensional mindful eating questionnaires, including the Mindful Eating Questionnaire (MEQ) (15) and the Four-Facets Mindful Eating Scale (FFaMES) (16). However, these tools exhibit certain limitations, such as the neglect of significant dimensions like non-judgment and issues related to the reliability and validity of the scales. In this context, Van Beekum et al. (5) developed and validated the Mindful Eating Scale (Mind-Eat Scale) in 2024. This scale was constructed using a representative sample of 3,102 individuals from the French NutriNet-Santé cohort. Through exploratory factor analysis, six dimensions were identified: awareness, non-reactivity, openness, gratitude, non-judgment, and hunger/satiety. Each dimension comprises four items, resulting in a total of 24 items. The dimensional framework of this scale is relatively comprehensive and demonstrates robust psychometric properties (5). Although the Mindful Eating Questionnaire (MEQ) has been previously translated into Chinese, it has notable limitations, including insufficient coverage of the ‘non-judgment’ and ‘gratitude’ dimensions, and relatively weak psychometric performance in certain subscales when applied to non-Western populations. In contrast, the newly developed Mind-Eat Scale offers a more comprehensive six-dimensional framework (including gratitude and non-reactivity) and was validated using a large, population-based cohort, suggesting superior content coverage and robustness. Therefore, introducing this scale to China fills a critical conceptual gap beyond simply providing another translated instrument.

The core concept of mindful eating aligns well with traditional Chinese dietary culture. Chinese traditional culture emphasizes practices such as “avoiding conversation during meals”, “chewing slowly”, and “eating until seven-tenths full”, which closely correspond to the dimensions of mindful eating, including awareness and hunger/satiety. However, cultural factors significantly influence an individual’s dietary behaviors, food preferences, and interpretations of the concept of “mindfulness”. Directly applying Western scales may introduce cultural biases, necessitating systematic cross-cultural adjustments and validations. Currently, there is a lack of effective mindful eating assessment tools specifically designed for the Chinese adults, and no studies have systematically localized the Mind-Eat Scale or conducted psychometric evaluations within this demographic. Therefore, this study aims to localize and cross-culturally adapt the Mind-Eat Scale and systematically assess its psychometric characteristics in the Chinese general population. Ultimately, we aspire to provide a validated tool for assessing the level of mindful eating among the Chinese general population, establishing a foundation for epidemiological studies that explore the relationships between mindful eating, dietary behaviors, weight status, mental health, and brain function. Additionally, this research will offer a basis for implementing mindful eating interventions in clinical nursing practice. The execution of this study will enrich the cross-cultural research evidence in the field of nutritional psychology and advance the scientific research and practical application of mindful eating strategies within the Chinese population.

## Methods

### Design and participants

This multicenter cross-sectional study was conducted in three communities across three provinces of China from January to March 2026: Jiangxi Province (southern China), Xinjiang Uygur Autonomous Region (western China), and Heilongjiang Province (northern China). A total of 346 adults were recruited using a convenience sampling method, with all participants randomly selected. The questionnaire survey was administered through the online data collection software “Wenjuanxing”, with participants completing the questionnaire via smartphones. The sample size for this study was determined based on the requirements for factor analysis, which stipulates that each item should have at least 10 participants (17). Given that the research scale comprises 24 items, a minimum of 240 participants was necessary. However, considering that confirmatory factor analysis requires a sample size of at least 200 cases (18), and that exploratory and confirmatory factor analyses should be conducted on different samples (19), the sample size was expanded to account for potential low response rates during data collection. Ultimately, 346 participants were successfully recruited. The inclusion criteria for participants were: (1) adults aged 18 years or older; and (2) voluntary participation after signing an informed consent form.

### Measures

#### Participant’s general demographic characteristics

After a thorough literature review and full discussion among the research team, the research members developed a questionnaire for the general demographic characteristics of the participants in this study. It consists of 8 questions, including age, sex, marital status, education level, current career status, whether you have had meditation or mindfulness practice experiences, whether you have ever deliberately restricted your diet to control your weight, etc.

#### Mindful Eating Scale

This scale was developed by Van Beekum et al. (5) in 2024 through a systematic four-step process. It comprises six dimensions: awareness, non-reactivity, openness, gratitude, non-judgment, and hunger/satiety. Each dimension includes four items, resulting in a total of 24 items. The scale utilizes a 5-point Likert scale for scoring, with responses ranging from 1 to 5, corresponding to ‘never,’ ‘rarely’, ‘sometimes’, ‘often’, and ‘always’. The minimum score on this scale is 24, while the maximum score is 120. A higher score indicates a greater level of mindfulness in eating. The Cronbach’s alpha coefficients for the six dimensions range from 0.84 to 0.88, reflecting strong reliability.

### Procedures

#### Translation and cultural adaptation

The researchers secured permission from Professor Van Beekum, the creator of the Mind-Eat Scale, by reaching out through email. They implemented Brislin’s translation model (20) to guide their efforts and rigorously followed the established adaptation and validation protocols necessary for cross-cultural research tools during the adaptation of the scale for a Chinese-speaking audience (21). To begin the process, two Chinese medical doctoral students, who were also fluent in English, took on the task of translating the English version of the scale into two separate Chinese translations. Following this, an independent medical doctoral student who had not participated in the initial translation effort compared the two Chinese renditions and synthesized them into a single preliminary draft of the Chinese version. The next step involved two Chinese professors, each proficient in English, who conducted reverse translations of the preliminary draft to ensure accuracy and fidelity to the original scale. The translated version then underwent further scrutiny and refinement by four experts in public health and three experts in psychology, utilizing the Delphi expert consultation method (21). All the experts involved held senior or higher professional titles, possessed at least a master’s degree, and willingly engaged in this study out of professional interest and expertise. Based on the feedback provided by these experts, the scale was revised accordingly. The researchers performed a critical review of the initial draft of the Chinese version, focusing specifically on achieving semantic, content, and conceptual equivalence with the English original. Additionally, they addressed any controversial items and made necessary language and cultural modifications to ensure that the scale’s content resonated appropriately within the Chinese medical context and linguistic landscape. Before the launch of the formal survey, a preliminary study was conducted with a sample of 20 adults, who were subsequently excluded from participating in the main survey. Data collection occurred online via the platform “Wenjuanxing”^1^, enabling participants to complete the questionnaire conveniently using their smartphones. Participants indicated that the scale items were clear and comprehensible, with the questionnaire taking an estimated 4 to 5 min to complete. This comprehensive process culminated in the final version of the Chinese Mind-Eat Scale.

### Data collection

Data collection for this study was carried out utilizing the ‘Wenjuanxing’ software through an online approach. To initiate this process, the researchers first reached out to the leaders of three distinct communities, engaging in comprehensive discussions to build rapport and establish trust. This foundational communication was essential, as it set the stage for a collaborative relationship between the research team and the community heads, ensuring a smoother data collection process. During the questionnaire distribution phase, the community heads made themselves readily available to the researchers, allowing for immediate support should any issues or questions arise. They were initially briefed on the study’s inclusion and exclusion criteria, which facilitated the effective screening of potential participants. Once the criteria were understood, the community heads were responsible for disseminating the questionnaire link—created by the research team—to all members of their respective communities in advance of the survey. The introduction page of the questionnaire was structured to provide comprehensive information regarding the research objectives, measures undertaken to ensure data confidentiality, and the rights of participants, including the option to withdraw from the study at any point without facing any consequences. This emphasis on informed consent was crucial, as it ensured that participants were fully aware of their rights before voluntarily completing the online questionnaire. To ensure data quality, we employed three complementary criteria for identifying invalid responses: (1) completion time of less than 4 min (based on the pilot test average of 4.5 min); (2) long-string analysis, defined as ≥10 consecutive identical responses on Likert-scale items; and (3) semantic consistency check using two pairs of negatively/positively worded items. Questionnaires meeting any of these criteria were excluded. Using these combined criteria, we excluded an additional 4 questionnaires, yielding a final valid sample of 346. Ultimately, a total of 350 questionnaires were distributed, and following the exclusion of invalid responses, the research team successfully obtained 346 valid questionnaires. This resulted in an impressive effective response rate of 98.9%, highlighting the efficacy of the data collection process.

### Data analysis procedure

The analysis of data was conducted utilizing SPSS version 25.0 alongside Amos version 24.0. Descriptive statistics were employed to examine the demographic features of the participants. Frequencies and percentages were used to represent categorical variables, whereas continuous variables were described as mean ± standard deviation (SD). A statistical significance was determined at *p* < 0.05.

### Items analysis

The analysis of items was conducted using three methods: the critical ratio, the correlation coefficient, and the Cronbach *α* coefficient, to assess the Chinese version of the scale. Detailed procedures for each method are outlined as follows: (1) Critical Ratio Method: The overall score for each questionnaire was computed and then ranked from the lowest to highest. Participants were categorized into the top 27% (high score group) and the bottom 27% (low score group) to perform a two-independent-sample *t*-test, which evaluated the scale’s capacity for discrimination. A critical ratio of each item that is ≥3 and a *p*-value <0.05 suggest that the item discriminates appropriately (22, 23). (2) Correlation Coefficient Method: The relationship between the scores of individual items and the total score was assessed using Pearson correlation, which helps determine the homogeneity of the scale items. Generally, a correlation coefficient of ≥0.4 between individual item scores and the total scale score indicates that the scale maintains suitable homogeneity (22). (3) Cronbach *α* Coefficient Method: Following the removal of certain items, the Cronbach’s *α* coefficient was calculated. It is suggested that if the removal of an item leads to an increase in the Cronbach’s *α* coefficient of the scale, this implies that the item should be excluded (24).

### Reliability analysis

Reliability serves as a metric for evaluating both the accuracy and consistency of responses generated by a measuring instrument, providing insight into the true extent of the characteristic being evaluated (25, 26). In our investigation, we utilized Cronbach’s *α* coefficient, split-half reliability, and test–retest reliability to measure the internal consistency of the scale’s Chinese version. We suggest that the Chinese version’s reliability can be considered acceptable when the values for Cronbach’s *α* coefficient, split-half reliability, and test–retest reliability each reach or exceed 0.70 (27, 28).

### Validity analysis

This research examined the scale’s validity from two angles: content validity and structural validity. Content validity is defined as the degree to which the items in the research instrument accurately represent the content being assessed. In this investigation, the Delphi method was utilized to involve four experts in public health and three in psychology to assess the questionnaire’s content validity. Expert feedback was collected through a 4-point Likert rating scale, where scores ranged from 1 (not relevant) to 4 (highly relevant). Using the ratings provided by the experts, content validity was sequentially assessed at both the item level (I-CVI) and the scale level (S-CVI). The calculation of I-CVI follows this formula: the number of experts giving a rating of 3 or higher divided by the total number of experts, with a threshold value exceeding 0.78 (28, 29). S-CVI is derived as the mean of the I-CVI values across the 24 items, and an S-CVI of 0.90 or higher indicates that the content validity of the translated scale is deemed acceptable (28, 29).

This research utilized exploratory factor analysis (EFA) and confirmatory factor analysis (CFA) to evaluate the structural validity of the scale. Using SPSS version 25.0, a total of 346 participants were randomly allocated into two distinct groups: one consisting of 136 individuals for EFA and the other comprising 210 participants for CFA. EFA was carried out to investigate the possible factor structure and assess its consistency with theoretical expectations. A key condition for the applicability of the translated scale in factor analysis is that the Kaiser-Meyer-Olkin (KMO) statistic must be above 0.60, and there should be significant results in Bartlett’s test of sphericity (*p* < 0.05) (30). Principal component analysis (PCA) along with maximum variance orthogonal rotation (MAVOR) were utilized to derive eigenvalues, factor loadings, and contribution rates, as well as to produce scatter plots, which facilitated the evaluation of the appropriateness of item configurations and dimension allocations for the Chinese version of the scale. The established criteria for exploratory factor items include (30): (1) Factor loading for each item needs to be at least 0.4, without any cross-loadings; (2) Every common factor is required to include a minimum of three items; (3) All common factors should collectively account for at least 60% of the total variance. CFA was conducted with Amos software version 24.0 to analyze the model’s fit indices. It is suggested that a model is considered a good fit if the chi-square degrees of freedom (CMIN/DF) is 3.0 or less, the approximate root mean square error (RMSEA) is 0.08 or lower, the comparative fit index (CFI) is 0.90 or higher, the Tucker-Lewis index (TLI) is 0.90 or above, the incremental fit index (IFI) is 0.90 or greater, and the Parsimonious normed-of-fit index (PNFI) is 0.7 or more (29, 31).

## Results

### Sociodemographic characteristics of the participants

This study involved a total of 346 participants, comprising 123 males and 223 females. Among the participants, 82.08% were aged between 18 and 39 years, while 70.23% were unmarried. Additionally, 74.86% held an undergraduate degree, and 54.91% identified as students. Notably, 74.86% of the participants had never engaged in mindfulness meditation or similar practices, and 46.24% had never intentionally restricted their diet for weight control. Furthermore, 50% reported a moderate level of overall physical activity in the past week. For comprehensive details regarding the sociodemographic characteristics of the participants, please refer to Table 1.

**Table 1:** Sociodemographic characteristics of the participants (*n* = 346).

Factors	Group	*n*	%
Age	18–39	284	82.08
	40–59	49	14.16
	>60	13	3.76
Sex	Male	123	35.55
	Female	223	64.45
Marital status	Unmarried	243	70.23
	Married	99	28.61
	Divorced or widowed	4	1.16
Education level	Primary school and below	9	2.60
	Technical secondary school	23	6.65
	Jounior college education	20	5.78
	Undergraduate education	259	74.86
	Postgraduate education	35	10.12
Current career status	On-the-job	122	35.26
	Students in school	190	54.91
	Retired	14	4.05
	Unemployed/underemployed	5	1.45
	Others	15	4.34
Have you ever engaged in any meditation or mindfulness practices?	Never practiced	259	74.86
	Practiced before (but have stopped now)	47	13.58
	Occasionally practice now (a few times a month)	34	9.83
	Regularly practice now (once a week)	6	1.73
Have you ever deliberately restricted your food intake in an effort to control your weight?	Never	160	46.24
	Had in the past but no longer now	137	39.60
	Currently doing so	49	14.16
Based on the situation over the past week, what is your level of physical activity?	Low (mainly sedentary, with almost no regular exercise)	158	45.66
	Moderate (regular moderate-intensity exercise, such as brisk walking, cycling, at least 3 days a week)	173	50
	High (regular high-intensity exercise, such as running, swimming, at least 3 days a week or engaging in heavy physical activities)	15	4.34

### The translation and revision of the scale

After a rigorous translation process, we invited four public health experts and three psychology experts to revise the 24-item Chinese version of the Mind-Eat Scale draft. Based on the results of the Delphi expert consultation survey, the recovery rate of the expert consultation questionnaire, the authority coefficient of the experts, and Kendall’s concordance coefficient were 1.000, 0.936, and 0.743, respectively. To enhance the scale items’ suitability for the Chinese context and expression habits, we revised three items sequentially. We modified the original item 6 from “I try new foods, even if I have a preconceived negative opinion about them” to “Even if I had a negative impression of certain foods before, I would still try them,” making the expression more neutral and aligning it with colloquial Chinese. We revised item 16 from “When I eat, I feel grateful for the planet that has provided me with food” to “While having my meal, I was grateful for the fact that this food was hard-won,” which better reflects the Chinese cultural context and enhances cultural appeal. We also changed item 24 from “When I eat, I think of the people or elements that produced my food (farmers, rain, animals, etc.)” to “When having meals, I will think about the people who worked hard to produce these foods and the natural conditions”, improving the fluency and formality of the sentence. The revised items align more closely with Chinese expression habits and are easier to understand. Following a preliminary survey using the revised version of the Mindful Eating Scale draft, all 20 participants indicated that each item was easy to understand and answer. The final development of the Chinese version of the Mind-Eat Scale standard prediction version is now complete, comprising 24 items.

### Items analysis

The quality of the items was evaluated through items analysis, which included the critical ratio method, item-scale correlation coefficients, and Cronbach’s *α* coefficient. The results indicated that the differences between the high-scoring and low-scoring groups were statistically significant for all items (*p* < 0.001). The critical ratios for the 24 items ranged from 6.511 to 15.879 (*p* < 0.001). The correlation coefficients between each item and the total score varied from 0.474 to 0.743 (*p* < 0.001). A comparison of the scale reliability after the deletion of each item from item 1 to item 24 revealed that the Cronbach’s *α* coefficients ranged from 0.922 to 0.927, all of which were lower than the Cronbach’s *α* coefficient of the total scale. Detailed information is presented in Table 2.

**Table 2:** Items analysis for Chinese version of the Mindful Eating Scale.

Item	Critical ratio	Correlation cofficient between item and total score	Cronbach’s Alpha if item deleted
Non-reactivity-1	7.795	0.554	0.925
Awareness-1	9.164	0.569	0.925
Gratitude-1	12.116	0.608	0.924
Non-judgement-1	8.492	0.474	0.927
Awareness-2	11.839	0.661	0.923
Openness-1	6.511	0.497	0.926
Hunger/satiety-1	8.913	0.568	0.925
Awareness-3	9.909	0.634	0.924
Openness-2	8.231	0.580	0.924
Non-judgement-2	7.187	0.495	0.926
Gratitude-2	13.324	0.681	0.923
Hunger/satiety-2	9.253	0.585	0.924
Openness-3	12.182	0.665	0.923
Non-reactivity-2	14.940	0.722	0.922
Hunger/satiety-3	7.208	0.520	0.925
Gratitude-3	12.806	0.642	0.923
Non-judgement-3	9.041	0.528	0.926
Non-reactivity-3	15.879	0.743	0.922
Hunger/satiety-4	11.301	0.675	0.923
Awareness-4	12.384	0.691	0.922
Openness-4	11.995	0.681	0.923
Non-judgement-4	9.780	0.588	0.924
Non-reactivity-4	14.231	0.736	0.922
Gratitude-4	11.815	0.639	0.923

### Reliability analysis

The Cronbach’s *α* value for this scale is 0.927, indicating high internal consistency. The Cronbach’s *α* values for the six dimensions of the scale range from 0.757 to 0.864, further supporting the reliability of the instrument. Additionally, the split-half reliability of the scale is measured at 0.895. A follow-up retest was conducted 2 weeks later with 30 participants who had previously been assessed, yielding a test–retest reliability of 0.862 (Table 3).

**Table 3:** Reliability analysis for Chinese version of the Mindful Eating Scale.

The scale and its dimension	Cronbach’s Alpha	Split-half reliability	Test–retest reliability
The Mindful Eating Scale	0.927	0.895	0.862
Awareness	0.864		
Non-reactivity	0.839		
Openness	0.757		
Gratitude	0.835		
Non-judgement	0.838		
Hunger/satiety	0.821		

### Validity analysis

#### Content validity

Seven qualified experts in public health and psychology were invited to evaluate the I-CVI and the S-CVI. Following the Delphi expert consultation, the response rate among the experts was 100%. The I-CVI values ranged from 0.857 to 1.000, while the S-CVI was recorded at 0.964 (Table 4).

**Table 4:** Content validity analysis for Chinese version of the Mindful Eating Scale.

Items	Expert 1	Expert 2	Expert 3	Expert 4	Expert 5	Expert 6	Expert 7	I-CVI	S-CVI
1	4	4	4	4	4	4	4	1.000	0.964
2	4	4	4	4	4	4	4	1.000	
3	4	4	4	4	4	4	4	1.000	
4	4	4	3	4	3	4	2	0.857	
5	4	4	4	3	3	3	4	1.000	
6	4	4	4	3	3	2	3	0.857	
7	4	4	4	3	3	2	3	0.857	
8	3	4	4	4	4	4	4	1.000	
9	4	4	3	2	3	4	4	0.857	
10	4	4	2	3	4	4	3	0.857	
11	4	4	4	4	4	4	4	1.000	
12	4	4	4	4	3	3	3	1.000	
13	4	4	4	4	3	3	3	1.000	
14	3	3	3	3	3	4	4	1.000	
15	4	4	4	4	4	2	3	0.857	
16	4	3	4	4	4	4	3	1.000	
17	4	4	4	4	4	4	4	1.000	
18	4	4	4	4	4	4	4	1.000	
19	4	4	4	4	4	4	4	1.000	
20	4	4	3	4	4	4	4	1.000	
21	4	4	4	4	4	4	4	1.000	
22	4	4	4	3	3	4	4	1.000	
23	4	4	4	4	3	4	3	1.000	
24	4	3	4	4	4	4	4	1.000	

#### Structural validity

##### Exploratory factor analysis

**Figure 1:**
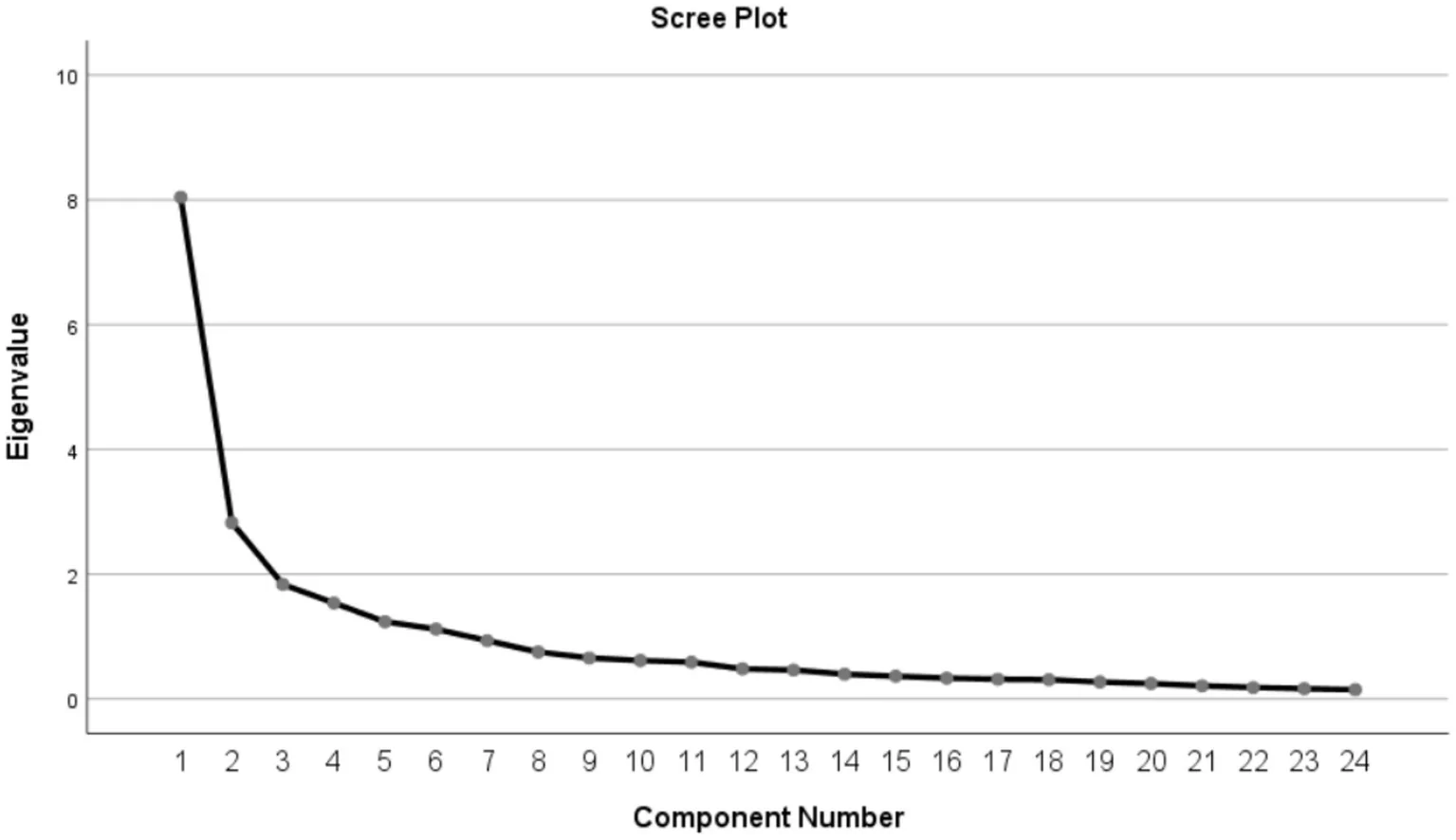
Screen plot of exploratory factor analysis for Chinese version of the Mindful Eating Scale.

Before conducting the EFA, Sample A (*n* = 136) underwent a test for factor model adequacy. In this study, the KMO measure yielded a value of 0.848, and the Bartlett’s Test of Sphericity was statistically significant (χ^2^ = 1757.176; *p* < 0.001), indicating that factor analysis was appropriate. The principal component analysis method was employed to extract six factors with eigenvalues greater than 1, which collectively accounted for 69.135% of the total variance. The scree plot (Figure 1) further supported the existence of a six-factor model, as the curve exhibited a diminishing trend after the sixth point. Additionally, the factor loadings for each item exceeded 0.4, and no items displayed double or multiple loadings (Table 5). Each factor contained at least three items, and none were removed from the analysis.

**Table 5:** Factor loadings of exploratory factor analysis for Chinese version of the Mindful Eating Scale.

Item	Factor 1	Factor 2	Factor 3	Factor 4	Factor 5	Factor 6
Non-reactivity-1	–	–	–	–	–	0.721
Awareness-1	–	0.661	–	–	–	–
Gratitude-1	0.670	–	–	–	–	–
Non-judgement-1	–	–	–	–	0.796	–
Awareness-2	–	0.774	–	–	–	–
Openness-1	–	–	–	0.688	–	–
Hunger/satiety-1	–	–	0.819	–	–	–
Awareness-3	–	0.821	–	–	–	–
Openness-2	–	–	–	0.776	–	–
Non-judgement-2	–	–	–	–	0.713	–
Gratitude-2	0.667	–	–	–	–	–
Hunger/satiety-2	–	–	0.780	–	–	–
Openness-3	–	–	–	0.653	–	–
Non-reactivity-2	–	–	–	–	–	0.529
Hunger/satiety-3	–	–	0.596	–	–	–
Gratitude-3	0.762	–	–	–	–	–
Non-judgement-3	–	–	–	–	0.758	–
Non-reactivity-3	–	–	–	–	–	0.728
Hunger/satiety-4	–	–	0.715	–	–	–
Awareness-4	–	0.675	–	–	–	–
Openness-4	–	–	–	0.680	–	–
Non-judgement-4	–	–	–	–	0.768	–
Non-reactivity-4	–	–	–	–	–	0.551
Gratitude-4	0.785	–	–	–	–	–

##### Confirmatory factor analysis

**Figure 2:**
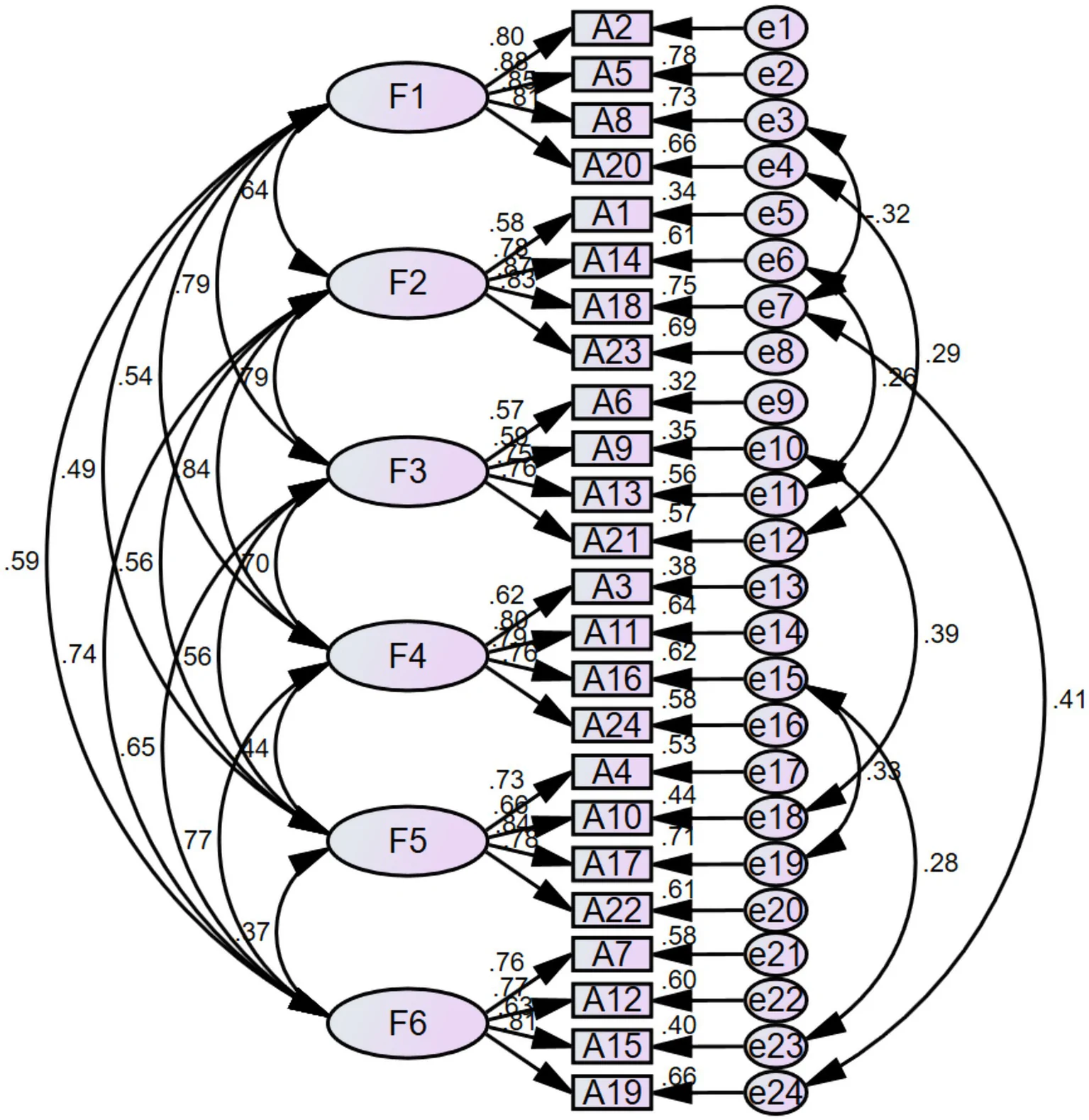
Standardized six-factor model for Chinese version of the Mindful Eating Scale (*n* = 210). F1: Awareness; F2: Non-reactivity; F3: Openness; F4: Gratitude; F5: Non-judgement; F6: Hunger/satiety.

In theCFA, the SEM was employed using the maximum likelihood method to fit the six-factor model (Figure 2). Based on the modification indices, the initial model underwent six revisions, specifically: e10 with e18, e7 with e24, e4 with e12, e15 with e23, e15 with e19, and e3 with e7. The final fitting results of the CFA model were as follows: χ^2^/df = 2.273, RMSEA = 0.078, TLI = 0.908, CFI = 0.904, IFI = 0.906, and PNFI = 0.706. All fitting indices indicated that the six-factor structure scale exhibited a suitable fit, further corroborating its alignment with the results of the EFA.

## Discussion

Mindful eating, recognized as a significant behavioral strategy for promoting healthy dietary habits and enhancing mental health, has garnered considerable international attention in recent years (3, 32, 33). However, there has been a persistent lack of effective measurement tools for assessing mindful eating within Chinese adults, which has somewhat hindered the advancement of empirical research in this domain in China. This study introduces the Mind-Eat Scale, developed by Van Beekum et al. (5) in 2024, into China for the first time and systematically evaluates its psychometric properties in the Chinese adults. The validation and verification of the Chinese version of the Mind-Eat Scale address the existing research gap regarding assessment tools for mindful eating in this population.

### The Chinese version of the Mindful Eating Scale has appropriate validity

Validity refers to the extent to which a measurement tool accurately assesses the intended construct (24). This study conducted validity tests on the Chinese version of the Mindful Eating Scale from two perspectives: content validity and construct validity. Content validity assesses the representativeness of the scale items concerning the measured content (28). We invited four public health experts and three psychology experts to systematically evaluate the scale items using the Delphi expert consultation method. The expert active coefficient was 100%, the expert authority coefficient was 0.936, and the Kendall harmony coefficient was 0.743, indicating strong consistency and reliability among the experts’ opinions. The I-CVI ranged from 0.857 to 1.000, and the S-CVI was 0.964, both exceeding the recommended standards (28, 29). These results indicate that the Chinese version of the Mindful Eating Scale possesses good content validity, with each item effectively reflecting the core connotation of mindful eating.

Construct validity refers to the extent to which a scale accurately measures the theoretical construct it purports to assess (30). In this study, we employed a combination of EFA and CFA to evaluate the construct validity of the scale. The EFA results indicated a KMO value of 0.848, and the Bartlett’s test of sphericity was statistically significant (χ^2^ = 1757.176, *p* < 0.001), confirming that the data were appropriate for factor analysis. Principal Component Analysis revealed six factors with eigenvalues greater than 1, resulting in a cumulative variance explanation rate of 69.135%, surpassing the recommended threshold of 60% (30). The factor loadings for all items exceeded 0.4, with no instances of cross-loading observed. All items were aligned with the six dimensions anticipated by the theoretical framework. CFA provided further validation of the six-factor structure, with all model fit indices meeting acceptable criteria (29). These findings suggest that the Chinese version of the Mind-Eat Scale maintains structural consistency with the original scale, and the six dimensions were validated within Chinese adults.

Notably, the ‘Gratitude’ dimension demonstrated particularly strong psychometric performance in our sample (Cronbach’s *α* = 0.835, factor loadings ranging from 0.667 to 0.785). We attribute this to traditional Chinese cultural values that emphasize ‘cherishing food’ (惜食) and respect for agricultural labor. Chinese cultural proverbs such as ‘every grain on the plate is the fruit of hard labor’ (谁知盘中餐, 粒粒皆辛苦) are widely taught from childhood, potentially making the concept of gratitude toward food sources more salient and easily endorsable for Chinese respondents compared to Western populations.

While the factor loadings for each dimension in this study were largely consistent with those of the original study (5), some items, particularly those related to the Non-reactivity dimension, exhibited slightly lower factor loadings than reported in the original study. We propose a cultural interpretation: in collectivist Chinese culture, self-restraint in front of desirable foods is often externally imposed by social norms (e.g., table manners, politeness) rather than stemming from internal mindful awareness. This external versus internal locus of behavioral regulation may lead to different interpretations of non-reactivity items among Chinese young adults, potentially attenuating the factor loadings.

Comparing our six-factor structure with the original French validation by Van Beekum et al. (5), we found substantial consistency in the overall dimensional framework, supporting the cross-cultural stability of the Mind-Eat Scale’s theoretical structure. However, the ‘Non-reactivity’ dimension showed slightly weaker loadings in our sample, suggesting potential cultural moderation. This pattern aligns with previous cross-cultural mindfulness research indicating that the expression and experience of mindfulness-related constructs may be influenced by cultural dimensions such as individualism–collectivism.

### The Chinese version of the Mindful Eating Scale has good reliability

Reliability is defined as the degree of consistency and stability exhibited by a measurement instrument (25, 26). This research assessed the reliability of the Mind-Eat Scale in its Chinese adaptation by employing Cronbach’s *α* coefficient, split-half reliability, and test–retest reliability. The overall Cronbach’s *α* coefficient for the scale was found to be 0.927, with coefficients for individual dimensions ranging between 0.757 and 0.864, all of which surpassed the acceptable minimum of 0.70 (27). This result is in close alignment with findings from the original research (5). In terms of split-half reliability, the coefficient recorded was 0.895, exceeding the recommended level of 0.70 (27), indicating strong consistency among the items. For the test–retest aspect, we conducted a re-evaluation of 30 participants following a two-week period, resulting in a coefficient of 0.862, once more above the accepted threshold of 0.70 (27). This outcome implies that the Chinese Mind-Eat Scale exhibits excellent stability over time, which supports its use at various intervals. To summarize, the Chinese version of the Mind-Eat Scale shows significant reliability.

### Limitations and prospects

This study acknowledges several limitations. Firstly, the sample sources are restricted to three communities across three provinces in China, representing the southern, western, and northern regions. While these areas are included, the overall representativeness of the sample requires enhancement. Furthermore, the sample predominantly consists of young adults (82.08% aged 18–39) and highly educated individuals (74.86% with undergraduate degrees), which limits the generalizability of our findings. Mindful eating is particularly relevant to clinical populations such as individuals with obesity, diabetes, and binge eating disorders. Future validation studies should intentionally recruit these populations and include older adults, individuals with lower educational backgrounds, and rural populations to establish the scale’s applicability across the full spectrum of the Chinese population. Secondly, the self-report nature of the questionnaire utilized in this study introduces potential reporting biases that are challenging to eliminate entirely. Participants may be influenced by social expectations, leading to biases in their self-evaluation of mindful eating levels. Future studies could integrate methodologies such as behavioral observation and ecological momentary assessment to yield more objective evaluations of mindful eating. Thirdly, certain items within the Non-reactivity dimension exhibited relatively low factor loadings. Future research should further investigate the stability of the factor structure for this dimension across various populations in China and make necessary optimizations and adjustments to the items. Fourthly, due to the limited sample size, we were unable to conduct multi-group measurement invariance testing across different demographic groups (e.g., age, sex, region). Future studies with larger and more diverse samples should examine measurement invariance to ensure the scale functions equivalently across different subgroups of the Chinese population.

## Conclusion

This study successfully introduced the Mind-Eat Scale to China and conducted a systematic assessment of its psychometric characteristics among a community-based convenience sample of Chinese adults. The Chinese version of the Mindful Eating Scale comprises six dimensions: awareness, non-reactivity, openness, gratitude, non-judgment, and hunger/satiety, totaling 24 items. It demonstrates satisfactory psychometric properties—including good internal consistency, test–retest reliability, content validity, and construct validity—within this specific sample. The scale can serve as a preliminary instrument for assessing mindful eating levels in community-dwelling Chinese adults. However, further validation in more diverse populations (including older adults, clinical groups, and rural populations) is warranted before broader application.

## Data Availability

The raw data supporting the conclusions of this article will be made available by the authors, without undue reservation.

## Footnotes

1
https://www.wjx.cn/
